# Assessing the risk of reoperation for mild pulmonary vein obstruction post-TAPVC repair: a retrospective cohort study

**DOI:** 10.3389/fcvm.2024.1399659

**Published:** 2024-06-26

**Authors:** Ailixiati Alifu, Haifan Wang, Renwei Chen

**Affiliations:** Department of Cardiothoracic Surgery, Hainan Women and Children’s Medical Center, Haikou, Hainan, China

**Keywords:** total anomalous pulmonary venous connection, reoperation, mild pulmonary vein obstruction, risk factor, retrospective cohort study

## Abstract

**Objective:**

This study investigates the impact of mild pulmonary vein obstruction, detected via echocardiography before hospital discharge, on the likelihood of reoperation in patients who have undergone repair for Total Anomalous Pulmonary Venous Connection (TAPVC).

**Method:**

Utilizing a single-center, retrospective cohort approach, we analyzed 38 cases from October 2017 to December 2023, excluding patients with functionally univentricular circulations or atrial isomerism. Our primary outcome was the necessity for reoperation within one year due to anatomical issues related to the initial TAPVC repair. Mild obstruction was defined as a pulmonary vein flow velocity ≥1.2 m/s.

**Result:**

Our findings revealed that 31.6% of patients exhibited pre-discharge mild obstruction. During the median follow-up of 10 months, reoperations were notably higher in the mild obstruction group compared to the normal group, with a significant association between pre-discharge mild obstruction and increased risk of reoperation. Specifically, in the fully adjusted model, mild obstruction was linked to a 13.9-fold increased risk of reoperation.

**Conclusion:**

Our results suggest that a pre-discharge echocardiography Doppler velocity threshold of 1.2 m/s could serve as a critical predictor for reoperation, emphasizing the need for targeted follow-up strategies for at-risk patients.

## Introduction

TAPVC is a congenital heart defect in which all pulmonary veins abnormally drain into the systemic veins, right atrium, or coronary sinus (1). Mortality rates following TAPVC repair have declined thanks to surgical advancements and more thorough preoperative assessments (2–4). However, despite these improvements, up to 20% of patients develop recurrent pulmonary venous (PV) obstruction or stenosis after initial TAPVC correction (5, 6). This frequent complication is linked to heightened morbidity and mortality risks, especially when additional surgical interventions are necessary to address the PV issues.

Current research acknowledges postoperative PV obstruction as a critical risk factor for adverse outcomes, including the need for reintervention and mortality (3, 5, 7). However, there is a notable variation in the criteria used to define obstruction (7). Specifically, the clinical significance of mild postoperative PV obstruction (Doppler velocity ≥1.2 m/s) remains under debate. The purpose of this study is to investigate if mild obstruction identified through echocardiogram before hospital discharge is a potential risk factor for reoperations following TAPVC repair. This will be achieved through a retrospective cohort study to assess the predictive value of this parameter in guiding postoperative care and improving risk assessment for TAPVC patients.

## Patients and methods

This is a single-center, retrospective cohort study, which has been approved by the Institutional Review Board of Hainan women and children's medical center. Written informed consent was waived by the Institutional Review Board of Hainan women and children's medical center due to the anonymous nature of the retrospective study. All the methods were carried out in accordance with the Helsinki Declaration guidelines. We retrospectively reviewed 43 consecutive cases who underwent repair TAPVC from October 2017 to December 2023 at Hainan women and children's medical center. TAPVC with functionally univentricular circulations or atrial isomerism were excluded.

### Date collection

The data collected during hospitalization for all patients included age, gender, weight, height, prenatal diagnosis, TAPVC type, coexisting cardiac anomalies (except atrial septal defects, ductus arteriosus), presence of other organ abnormalities, surgical approaches, mechanical ventilation, follow-up information after discharge, and perioperative discharge, and follow-up echocardiographic results.

### Study definition

The major outcome was the occurrence of unplanned surgical procedures on individual pulmonary veins (PVs), PV anastomosis, or PV confluence within 1 year post-discharge. Re-explorations for bleeding or delayed sternal closure were excluded from the count of reoperations. Mild PVO is defined as a condition where the average flow velocity exceeds 1.2 m/s. This measurement is taken using Doppler transthoracic echocardiography at the sites of pulmonary vein confluence, anastomosis, or in any individual pulmonary vein, prior to discharge after the repair of TAPVC. Transesophageal Doppler ultrasonography measuring a flow velocity exceeding 2 m/s in the pulmonary vein confluence, anastomosis, or any individual pulmonary vein, coupled with respiratory failure requiring mechanical ventilation support and heart failure, is an indication for further surgical intervention.

### Statistical analysis

Continuous variables were expressed as mean with standard deviation (SD) or median with interquartile range [M (P25, P75)] depending on distributional properties, with comparisons between groups conducted using the student's *t*-test or the Mann–Whitney *U*-test. Categorical variables were presented as absolute numbers and percentages, with comparisons between groups performed using Fisher's exact test.

Kaplan-Meier curves and 95% confidence intervals were utilized to estimate the event-free survival time distribution. Comparisons between groups were used the log-rank test. The association between pre-discharge mild obstruction and the risk of reoperation was analyzed using the Cox proportional hazards regression model. The proportional hazards assumption was satisfactory in all regression models. Initially, potential factors associated with reoperation were identified through univariate Cox regression analysis (*P* < 0.1), incorporating previous literature's evidence. Subsequently, we considered variables that were imbalanced between the pre-discharge mild obstruction and the normal group at baseline (Standard Mean Difference >0.2). Finally, because of severe multicollinearity between weight and age (Kappa = 111.25, VIF for weight = 43.33), we selected age, sex, performed prenatal diagnosis, TAPVC type, Preoperative-postoperative mechanical ventilation time as potential covariates. Using a hierarchical modeling approach, four models were constructed: (1) the unadjusted model; (2) Model 1 adjusted for age, sex, and TAPVC type; (3) Model 2 added prenatal diagnosis and preoperative mechanical ventilation based on Model 1; (4) Model 3, the fully adjusted model, added the duration of postoperative mechanical ventilation based on Model 2.

We conducted the threshold analysis for the pre-discharge echocardiographic Doppler velocity. A restricted cubic spline (RCS) plot, with 3 knots, was used to illustrate the nonlinear association between Doppler velocity and the risk of reoperation. Fully adjusted Cox regression model with the RCS term was employed in this process (using RMS package). Based on the results of the RCS plot, we used the multivariate Cox regression model as the sensitivity analyses, with the threshold of Doppler velocity ranging from 1 m/s to 1.6 m/s (intervals of 0.1 m/s). All statistical analyses were performed using R software (version 4.3.2). A *P*-value < 0.05 was considered statistically significant.

## Result

In this retrospective cohort study, 38 cases of TAPVC were analyzed, with 5 cases excluded (3 with functionally univentricular circulations, 2 with atrial isomerism). The breakdown included 16 supracardiac, 12 cardiac, 6 infracardiac, and 4 mixed types. The majority were males (65.8%), with a median age of 0.47months and weight of 3.12 kg. Notably, postoperative obstruction occurred in a total of 12 cases (31.6%) after TPAVC, with 5 cases (31.3%) being of the supracardiac type, 2 cases (16.7%) of the intracardiac type, 3 cases (50.0%) of the infracardiac type, and 2 cases (50.0%) of the mixed type.

Imbalanced baseline characteristics between the normal group and the pre-discharge mild obstruction group included age, sex, weight, prematurity, TAPVC type, preoperative ventilator assistance and duration of postoperative ventilator assistance. (Table 1) The median follow-up duration was 10.00 months [interquartile range: 5.12, 12.00], with a total of 297.1 patient-years. Five patients required reoperation. Among them, four underwent the procedure at our institution. One case, characterized as supracardiac type with right PVO, was successfully managed by employing a modified “sutureless,” free pericardial patch as described by Ricci (8). Furthermore, Gore-Tex patch enlargement was utilized in two cases of intracardiac restenosis at the coronary sinus-left atrium junction. Additionally, Gore-Tex enlargement anastomosis was performed in one case of infracardiac type obstruction. Moreover, one patient met the criteria for reoperation; however, they succumbed to ineffective resuscitation due to acute heart failure at an external facility.

**Table 1:** Baseline characteristics.

	Total, *n* = 38	Pulmonary vein flow velocity		*P*	SMD
		Normal, *n* = 26	Mild obstruction, *n* = 12		
Age, month	0.47 [0.27, 1.19]	0.60 [0.27, 3.04]	0.32 [0.22, 0.47]	0.105^a^	**0**.**293**
Sex, male	25 (65.8)	15 (57.7)	10 (83.3)	0.158	**0**.**493**
Weight, kg	3.12 [2.71, 3.84]	3.32 [2.96, 4.78]	2.58 [2.44, 3.03]	0.001^a^	**0**.**445**
Premature delivery, yes	3 (7.9)	1 (3.8)	2 (16.7)	0.229	**0**.**418**
Emergency visit, yes	17 (44.7)	12 (46.2)	5 (41.7)	1.000	0
Prenatal diagnosis, yes	7 (18.4)	4 (15.4)	3 (25.0)	0.656	0.154
Cardiac malformations, yes	12 (31.6)	9 (34.6)	3 (25.0)	0.714	0.126
Other malformations, yes	13 (34.2)	8 (30.8)	5 (41.7)	0.714	0.126
TAPVC subtype				0.396	**0**.**293**
Supracardiac	16 (42.1)	11 (42.3)	5 (41.7)		
Cardiac	12 (31.6)	10 (38.5)	2 (16.7)		
Infracardiac	6 (15.8)	3 (11.5)	3 (25.0)		
Mixed	4 (10.5)	2 (7.7)	2 (16.7)		
Preoperative mild Obstruction, yes	16 (42.1)	10 (38.5)	6 (50.0)	0.725	0.121
Preoperative ventilator assistance, yes	17 (44.7)	10 (38.5)	7 (58.3)	0.307	**0**.**354**
Duration of postoperative ventilator assistance, hours	75.11 ± 42.86	70.81 ± 46.85	84.42 ± 32.43	0.370^b^	**0**.**310**

Categorical variables were presented as *n* (%), *P*-value was calculated using Fisher's exact test; Continuous variables were presented as mean ± SD or median [interquartile range] as appropriate; ^a^Mann–Whitney *U*-test; ^b^Student *t-* test.

TRPVC, total anomalous pulmonary venous connection; SMD, standardized mean difference; SD, standard deviation.

Bold indicates SMD values >0.2.

**Figure 1:**
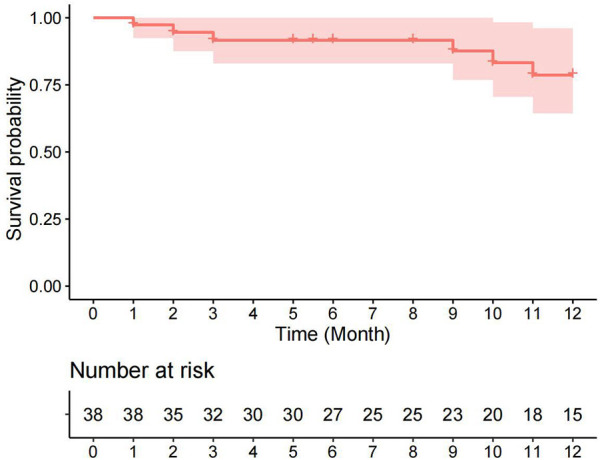
Kaplan-Meier curve (95% confidence interval) of survival without reoperation for the entire cohort.

**Figure 2:**
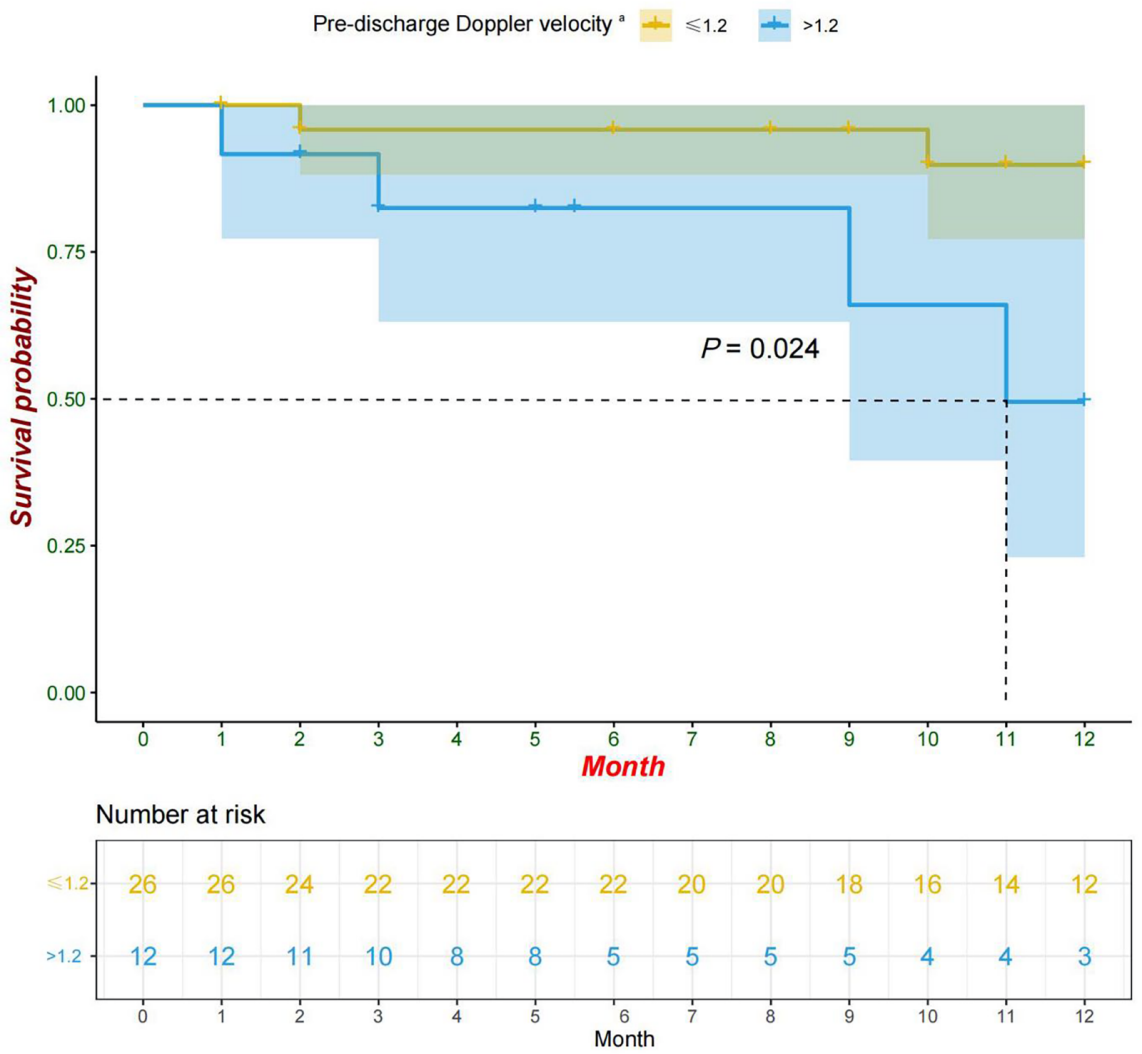
Kaplan-Meier curve (95% confidence interval) of survival without reoperation for the pre-discharge Doppler velocity ≤1.2 and >1.2 groups. ^a^Patients with pre-discharge echocardiographic Doppler velocity >1.2 were defined as mild obstruction in this study.

Univariate analysis revealed that the pre-discharge mild obstruction group had higher reoperation rates (33.3% vs. 7.7%; *P* = 0.066) compared to the normal group (Figure 1). Reoperations occurred within 3 months and between 9 and 11 months after discharge. The study reported survival rates of 91.6% at 6 months and 78.6% at 12 months. Kaplan-Meier analysis showed that the event-free survival rate of reoperation was significantly lower in the pre-discharge mild obstruction group compared to the normal group (*P* = 0.024) (Figure 2).

The results of the univariate Cox regression analysis show no statistically significant association (Table 2). Although, the findings suggest that each additional standard deviation in the duration of postoperative mechanical ventilation is associated with a higher risk of reoperation (HR = 1.80; 95% CI: 0.96–3.39; *P* = 0.069). The hierarchical multivariate Cox regression models show that pre-discharge mild obstruction was associated with reoperation (Table 3). In the unadjusted model, pre-discharge mild obstruction was associated with an increased risk of reoperation (HR = 5.75; 95% CI: 1.04–31.80; *P* = 0.045); In the fully adjusted model (Table 3—model 3), pre-discharge mild obstruction was associated with an approximately 13.9-fold increased risk of reoperation (HR = 13.90; 95% CI: 1.16–166.5; *P* = 0.038).

**Table 2:** Univariate cox regression analysis for the risk of reoperation.

Variable	HR (95%CI)	*P*
Age, months	0.96 (0.761–1.211)	0.729
Sex, male	2.18 (0.254–18.687)	0.477
Weight, kg	0.91 (0.610–1.351)	0.633
Premature delivery, yes	1.96 (0.228–16.804)	0.540
Emergency visit, yes	1.64 (0.329–8.160)	0.546
Prenatal diagnosis, yes	2.41 (0.440–13.149)	0.311
Cardiac malformation, yes	1.13 (0.206–6.195)	0.888
Other malformations, yes	1.15 (0.210–6.335)	0.871
TAPVC subtype, Supracardiac	Reference	
Cardiac	2.04 (0.281–14.778)	0.482
Infracardiac	2.11 (0.189–23.506)	0.544
Mixed	2.42 (0.218–26.803)	0.472
Preoperative mild obstruction, yes	0.67 (0.122–3.654)	0.642
Preoperative ventilator assistance, yes	2.98 (0.542–16.425)	0.209
Duration of postoperative ventilator assistance, per 1 SD increased	1.80 (0.96–3.39)	0.069

TRPVC, total anomalous pulmonary venous connection; HR, hazard ratio; CI, confidence interval; SD, standard deviation.

**Table 3:** Multivariable cox regression analysis of the association between pre-discharge mild obstruction and the risk of reoperation.

Variables	Model 1		Model 2		Model 3	
	HR (95%CI)	*P*	HR (95%CI)	*P*	HR (95%CI)	*P*
Pre-discharge mild obstruction, yes	6.80 (1.02–45.28)	0.047	7.67 (1.02–57.60)	0.048	13.90 (1.16–166.5)	0.038
Age, months	0.99 (0.92–1.08)	0.890	1.00 (0.93–1.08)	0.935	1.01 (0.94–1.09)	0.732
Sex, male	2.20 (0.18–27.06)	0.537	3.03 (0.24–38.58)	0.393	23.54 (0.35–1,580.6)	0.141
The type of TAPVC, supracardiac	1.00	Ref.	1.00	Ref.	1.00	Ref.
Cardiac	3.39 (0.40–29.17)	0.265	7.21 (0.47–111.70)	0.158	7.35 (0.42–128.6)	0.172
Infracardiac	2.41 (0.15–39.74)	0.539	2.40 (0.13–45.24)	0.558	4.19 (0.16–110.3)	0.390
Mixed	3.38 (0.26–44.25)	0.353	2.48 (0.17–35.31)	0.501	3.15 (0.18–55.63)	0.433
Prenatal diagnosis, yes			2.08 (0.24–18.25)	0.509	1.17 (0.10–14.32)	0.901
Preoperative ventilator assistance, yes			3.08 (0.36–26.13)	0.303	2.51 (0.20–31.47)	0.475
Duration of postoperative ventilator assistance, per 1 SD increased					1.04 (1.00–1.07)	0.063

TRPVC, total anomalous pulmonary venous connection; HR, hazard ratio; CI, confidence interval; SD, standard deviation.

**Figure 3:**
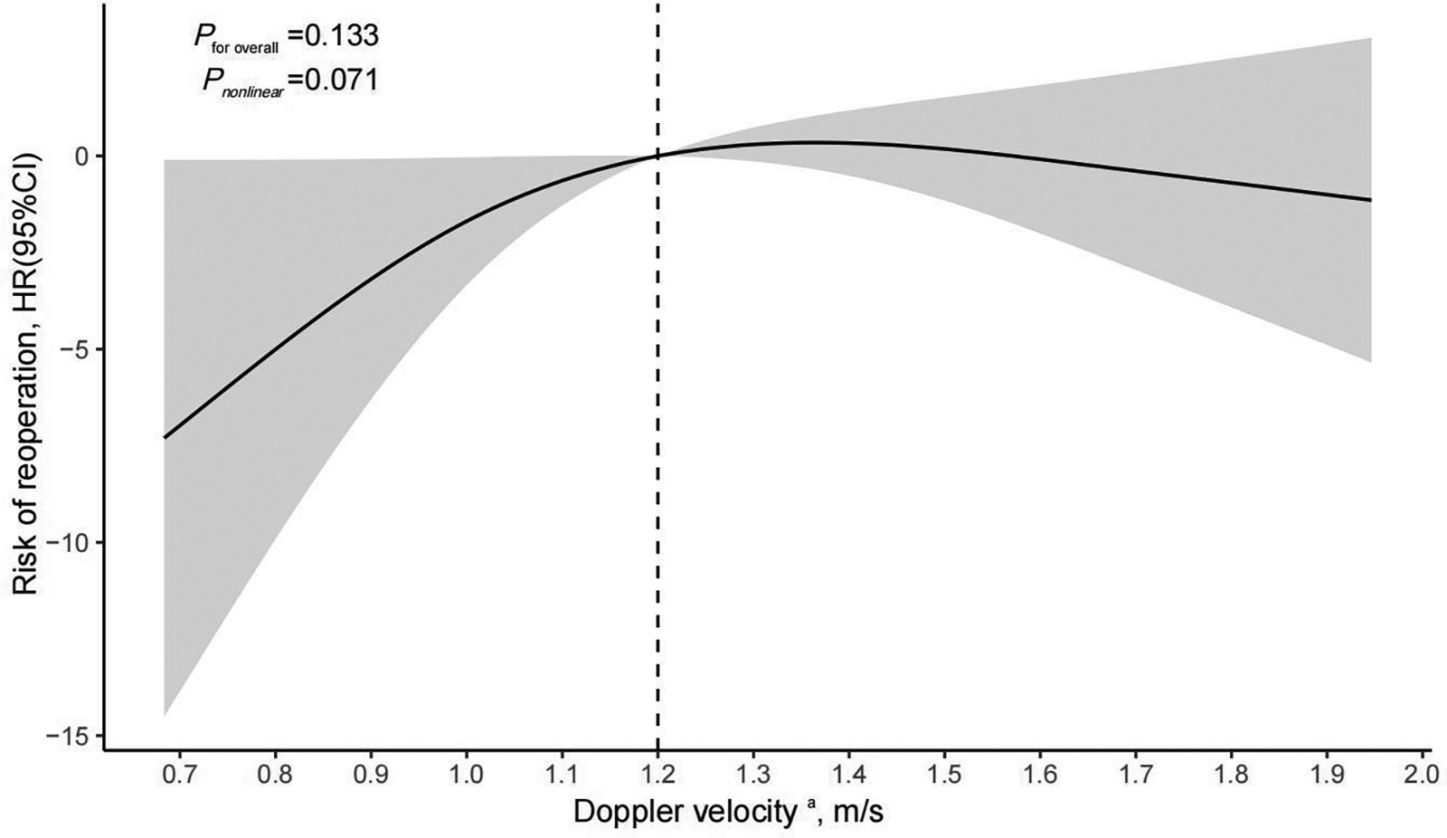
Cox regression models with restricted cubic splines were applied to examine association between pre-discharge echocardiographic Doppler velocity and risk of reoperation. ^a^Pre-discharge echocardiographic Doppler velocity were fitted as a non-linear term with the number of knots set to 3 and adjustment included age, sex, prenatal diagnosis, type of TAPVC, preoperative ventilator assistance, and duration of postoperative ventilator assistance; HR, hazard ratio; CI, confidence interval.

The analysis of restricted cubic splines suggests that there might be a nonlinear association between pre-discharge Doppler velocity by echocardiography and the risk of reoperation (Figure 3) although the test for nonlinearity was not statistically significant (*P* nonlinear = 0.071). As the Doppler velocity rising before approximately 1.2 m/s, there was a positive correlation between Doppler velocity and the risk of reoperation, and this association tended to plateau after exceeding 1.2 m/s. Multivariable Cox regression models, with different Doppler velocity thresholds, also indicated that 1.2 m/s is a key inflection point with the risk of reoperation corresponding to threshold velocities set at 1 m/s, 1.1 m/s, 1.2 m/s, 1.3 m/s, 1.4 m/s, 1.5 m/s, and 1.6 m/s being 3.38 (95% CI: 0.24–47.25, *P* = 0.365), 3.57 (95%CI: 0.25–49.95, *P* = 0.345), 13.9 (95%CI: 1.16–166.5, *P* = 0.038), 20.08 (95% CI: 0.72–563.2, *P* = 0.078), 10.98 (95% CI: 0.58–207.5, *P* = 0.110), 12.23 (95% CI: 0.71–211.5, *P* = 0.085), and 34.82 (95% CI: 0.59–2051.1, *P* = 0.088), respectively (Table 4). However, these analyses may be limited by insufficient statistical power due to low sample sizes in certain subgroups.

**Table 4:** Association between pre-discharge echocardiographic Doppler velocity with different threshold settings and the risk of reoperation.

Threshold setting	HR (95%CI)	*P*
Pre-discharge Doppler velocity >1	3.38 (0.24–47.25)	0.365
Pre-discharge Doppler velocity >1.1	3.57 (0.25–49.95)	0.345
Pre-discharge Doppler velocity>1.2	13.9 (1.16–166.5)	0.038
Pre-discharge Doppler velocity > 1.3	20.08 (0.72–563.19)	0.078
Pre-discharge Doppler velocity > 1.4	10.98 (0.58–207.52)	0.110
Pre-discharge Doppler velocity >1.5	12.23 (0.71–211.51)	0.085
Pre-discharge Doppler velocity >1.6	34.82 (0.59–2,051.06)	0.088

The models were adjusted for age, sex, prenatal diagnosis, type of TAPVC, preoperative ventilator assistance, and duration of postoperative ventilator assistance.

TRPVC, total anomalous pulmonary venous connection; HR, hazard ratio; CI, confidence interval.

## Discussion

Our study aimed to elucidate the impact of mild pulmonary vein obstruction, as detected by echocardiography before hospital discharge, on the risk of reoperation in patients who underwent repair for TAPVC. By focusing on a specific, quantifiable parameter (Doppler velocity ≥1.2 m/), we sought to reduce the subjectivity and variability inherent in diagnosing postoperative complications. This approach aligns with efforts to standardize postoperative assessments and could contribute to more uniform patient care protocols.

Our study's findings align with most of the research indicating that pulmonary vein obstruction is a risk factor for reoperation after TAPVC surgery (3, 5–7, 9–11). However, there is a consensus in the literature regarding the definition of obstruction, with the most accepted criteria being an echocardiography Doppler velocity of 2 m/s or more, a catheterization gradient of 4 mm Hg or more, or a narrowing of 50% or more by angiography (1, 12, 13). Despite this, various studies have employed different echocardiography cutoffs, including velocities of 1.2 m/s or more (7, 8), 1.5 m/s or more (10), and 1.8 m/s or more (14). It's noteworthy that many studies did not specify the criteria for determining PVO (11, 15, 16). Unlike these studies, which based their echocardiography findings on follow-up periods or before reoperation, our study's results are derived from echocardiograms performed before hospital discharge after TAPVC repair. This approach allows us to identify at-risk patients earlier, thereby emphasizing the importance of timely attention and the establishment of an earlier follow-up plan. In our center, children discharged from the hospital after TAPVC repair with cardiac echo flow velocities exceeding 1.2 m/s tended to receive more frequent follow-up within the first year post-discharge. Follow-up appointments include repeat echocardiograms at 1, 3, 6, and 12 months after discharge. This approach is based on findings from several studies indicating that reoperation rates are higher within the first year post-discharge (3, 6, 8, 15).

In this study, one patient who had undergone TAPVC surgery died during the post-discharge follow-up period. This case was included in the reoperation group because the last outpatient cardiac echocardiogram showed a pulmonary vein anastomosis flow velocity of 2.1 m/s, with no symptoms present, leading to a recommendation for continued close monitoring. However, during a later telephone follow-up, the parents reported that the child had experienced significant respiratory distress prior to death. The child was urgently transported to a local hospital, but unfortunately, resuscitation efforts were unsuccessful. This case met our criteria for further surgery.

The identification of a specific velocity threshold—1.2 m/s—as a critical predictor for reoperation within the first year post-discharge introduces a new and significant insight into postoperative care. Sengupta et al. developed a predictive model for assessing the risk of re-intervention in patients after repair surgery for TAPVC (6). This model includes a classification of residual lesions based on the mean gradient at the pulmonary veins and the left atrial anastomosis measured by echocardiography before discharge. A pressure difference of less than 2 mmHg is classified as Grade 1, 2–4 mmHg as Grade 2, and greater than 4 mmHg as Grade 3, with higher grades indicating a higher risk of re-intervention. Elevated flow velocities in the pulmonary vein often suggest obstructions or constrictions, which could arise from several factors including the surgical technique employed, the body's reaction to materials used during surgery, or pre-existing health conditions.

This study's strength lies in its focused examination of the predictive value of mild pulmonary vein obstruction, detected via echocardiography before hospital discharge, on the risk of reoperation in TAPVC repair patients. Rigorous statistical analysis was employed to mitigate bias, enhancing the credibility of the findings. While sutureless repair might theoretically lower restenosis rates, it is not commonly utilized for TAPVC repair at our hospital. Only three patients in our study underwent this procedure, which could potentially impact our results if sutureless repair proves to be beneficial. Additionally, the generalizability of our findings is constrained by the single-center, retrospective design, and the small sample size. These limitations may not fully capture the diversity of patient populations and surgical practices across different centers. Therefore, further multi-center studies with larger cohorts are necessary to validate these findings and explore their applicability to broader clinical settings. Such research endeavors will significantly contribute to enhancing our understanding of postoperative care for TAPVC patients.

## Conclusion

Our study highlights the potential significance of mild pulmonary vein obstruction, as detected by pre-discharge echocardiography, in predicting the need for reoperation among patients who have undergone TAPVC repair. These findings underscore the importance of early detection and close monitoring of this patient group. Identifying at-risk patients before discharge allows for targeted follow-up strategies, potentially reducing the incidence of severe complications and the need for subsequent surgical interventions. Future research should aim to confirm these findings in larger, multi-center cohorts and to explore the most effective monitoring and intervention strategies for patients with mild pulmonary vein obstruction post-TAPVC repair.

## Data Availability

The original contributions presented in the study are included in the article/Supplementary Material, further inquiries can be directed to the corresponding author.

## References

[1] Seale AN Uemura H Webber SA Partridge J Roughton M Ho SY Total anomalous pulmonary venous connection: morphology and outcome from an international population-based study. Circulation. (2010) 122:2718–26. 10.1161/CIRCULATIONAHA.110.940825 21135364

[2] Bando K Turrentine MW Ensing GJ Sun K Sharp TG Sekine Y Surgical management of total anomalous pulmonary venous connection. Thirty-year trends. Circulation. (1996) 94:II12–6. 8901712

[3] Karamlou T Gurofsky R Al Sukhni E Coles JG Williams WG Caldarone CA Factors associated with mortality and reoperation in 377 children with total anomalous pulmonary venous connection. Circulation. (2007) 115:1591–8. 10.1161/CIRCULATIONAHA.106.635441 17353446

[4] Bayya PR Varghese S Jayashankar JP Sudhakar A Balachandran R Kottayil BP Total anomalous pulmonary venous connection repair: single-center outcomes in a lower-middle income region. World J Pediatr Congenit Heart Surg. (2022) 13:458–65. 10.1177/21501351221103492 35757951

[5] Zhang H Shi G Chen H. Risk factors for postoperative pulmonary venous obstruction after surgical repair of total anomalous pulmonary venous connection: a systemic review and meta-analysis. Interact Cardiovasc Thorac Surg. (2022) 35(2):ivac162. 10.1093/icvts/ivac162 35713512 PMC9270848

[6] Sengupta A Gauvreau K Kaza A Baird CW Schidlow DN Del Nido PJ A risk prediction model for reintervention after total anomalous pulmonary venous connection repair. Ann Thorac Surg. (2023) 116:796–802. 10.1016/j.athoracsur.2022.05.058 35779604

[7] White BR Ho DY Faerber JA Katcoff H Glatz AC Mascio CE Repair of total anomalous pulmonary venous connection: risk factors for postoperative obstruction. Ann Thorac Surg. (2019) 108:122–9. 10.1016/j.athoracsur.2019.02.017 30885849 PMC6591098

[8] Ricci M Elliott M Cohen GA Catalan G Stark J de Leval MR Management of pulmonary venous obstruction after correction of TAPVC: risk factors for adverse outcome. Eur J Cardiothorac Surg (2003) 24:28–36. 10.1016/S1010-7940(03)00180-5 12853042

[9] Spigel ZA Edmunds EE Caldarone CA Hickey EJ Binsalamah ZM Heinle JS. Total anomalous pulmonary venous connection: influence of heterotaxy and venous obstruction on outcomes. J Thorac Cardiovasc Surg. (2022) 163:387–95.e3. 10.1016/j.jtcvs.2021.03.058 33966882

[10] Hoashi T Kagisaki K Kurosaki K Kitano M Shiraishi I Ichikawa H. Intrinsic obstruction in pulmonary venous drainage pathway is associated with poor surgical outcomes in patients with total anomalous pulmonary venous connection. Pediatr Cardiol. (2015) 36:432–7. 10.1007/s00246-014-1031-2 25274399

[11] Husain SA Maldonado E Rasch D Michalek J Taylor R Curzon C Total anomalous pulmonary venous connection: factors associated with mortality and recurrent pulmonary venous obstruction. Ann Thorac Surg. (2012) 94:825–31. 10.1016/j.athoracsur.2012.04.026 22633497

[12] Nakayama Y Hiramatsu T Iwata Y Okamura T Konuma T Matsumura G Surgical results for functional univentricular heart with total anomalous pulmonary venous connection over a 25-year experience. Ann Thorac Surg. (2012) 93:606–13. 10.1016/j.athoracsur.2011.09.038 22206962

[13] Hancock Friesen CL Zurakowski D Thiagarajan RR Forbess JM del Nido PJ Mayer JE Total anomalous pulmonary venous connection: an analysis of current management strategies in a single institution. Ann Thorac Surg (2005) 79:596–606. 10.1016/j.athoracsur.2004.07.005 15680843

[14] Shi G Zhu Z Chen J Ou Y Hong H Nie Z Total anomalous pulmonary venous connection: the current management strategies in a pediatric cohort of 768 patients. Circulation. (2017) 135:48–58. 10.1161/CIRCULATIONAHA.116.023889 27881562

[15] Karaci AR Harmandar B Aydemir NA Sasmazel A Balci AY Saritas T Early and intermediate term results for surgical correction of total anomalous pulmonary venous connection. J Card Surg. (2012) 27:376–80. 10.1111/j.1540-8191.2012.01435.x 22497245

[16] Kelle AM Backer CL Gossett JG Kaushal S Mavroudis C. Total anomalous pulmonary venous connection: results of surgical repair of 100 patients at a single institution. J Thorac Cardiovasc Surg. (2010) 139:1387–94.e3. 10.1016/j.jtcvs.2010.02.024 20392458

